# The second brain in autism spectrum disorder: could connexin 43 expressed in enteric glial cells play a role?

**DOI:** 10.3389/fncel.2015.00242

**Published:** 2015-07-03

**Authors:** Vladimir Grubišić, Vladimir Parpura

**Affiliations:** ^1^Department of Neurobiology, University of Alabama at BirminghamBirmingham, AL, USA; ^2^Neuroscience Program, Department of Physiology, Michigan State UniversityEast Lansing, MI, USA

**Keywords:** enteric nervous system, gastrointestinal motility, epithelial barrier, neurovascular unit, inflammation, Pitt-Hopkins syndrome, Rett syndrome

Autism spectrum disorder (ASD) is an umbrella term for a heterogeneous group of developmental disorders that present with persistent deficits in social communication/interaction and repetitive/restricted patterns of behavior, interests, or activities that cannot be better explained by intellectual disability or global developmental delay (DSM-5 APA, 2013). The recent estimate for ASD in 8-year old children in the United States is one in 68 (Baio and Autism and Developmental Disabilities Monitoring Network Surveillance Year 2010 Principal Investigators, 2014). Although a genetic cause can be identified in 20–25% cases (reviewed in Miles, 2011), the etiology of ASD still remains largely unclear. Several possible factors have been investigated to explain the etiology of ASD, i.e., immune dysregulation and inflammation, oxidative stress, mitochondrial dysfunction, and environmental factors (Rossignol and Frye, 2012).

Children with ASD have a variety of psychiatric, neurological, nutritional/metabolic, and other medical conditions (Venkat et al., 2012). One of the most common ASD comorbidities is a gut disorder, affecting 9–90% of ASD patients depending on the definitions and the groups studied (Buie et al., 2010). In comparison with typically developing children, general gastrointestinal (GI) symptoms are over five times more common in children with ASD; abdominal pain is reported more than twice as much and both constipation and diarrhea have four times higher incidence (McElhanon et al., 2014). Although gut disorders are not exclusive for ASD (Chaidez et al., 2014), there is a strong correlation of GI disturbances with autism severity (Adams et al., 2011). Abdominal pain could certainly worsen the behavior, especially in ASD patients that cannot express their discomfort due to speech impairment. Furthermore, recent findings point out to a more substantial association between the brain and the gut (Mayer et al., 2014).

Genetic mutations that cause impairment of the central nervous system (CNS) could directly affect the enteric nervous system (ENS), a nervous tissue within the gut wall often called “the second brain” due to its size, structure, complexity, and autonomic regulation of gut functions, such as bowel motility, secretion/absorption and local blood flow (Furness, 2006, 2012). For instance, a recent study reported reduced upper GI and distal colonic transit velocities in mice heterozygous for the deletion of *Transcription factor 4* (*TCF4*) (Grubišić et al., 2015), emulating symptoms, i.e., gastroesophageal reflux and constipation, of patients with the Pitt-Hopkins Syndrome (PTHS) (Whalen et al., 2012), a very rare ASD (Sweatt, 2013), caused by the haplo-insufficiency of the *TCF4* gene (Amiel et al., 2007; Brockschmidt et al., 2007; Zweier et al., 2007). Due to no obvious malformation of the gut in these animals (Grubišić et al., 2015) and the role of the basic helix-loop-helix transcription factors in neural crest development (Nelms and Labosky, 2010), we hypothesized that the reduced bowel motility in the above mouse model of PTHS was caused by the ENS dysfunction. Additionally, gut microbiota and GI barrier permeability were implicated as important etiological factors of ASD (Hsiao et al., 2013). Microbiota are locally important for maintaining the GI epithelial barrier (Kabouridis et al., 2015), while bacterial metabolites reaching the CNS can cause neuroinflammation and behavioral changes that resemble ASD (MacFabe et al., 2011). These findings indicate that GI disturbances, besides having an impact on the quality of life, can also contribute to the severity of neurological and psychiatric symptoms in ASD patients. Therefore, more research is needed to pinpoint the role of the second brain in ASD.

In recent years, enteric glial cells (EGCs), traditionally considered as only supportive cells of the ENS, are emerging as local GI regulators. They are strategically located at the interface between the neurons and other non-neuronal cells in the gut, such as enterocytes or immune cells, and participate in the regulation of gut motility, intestinal epithelial barrier, and inflammatory processes (reviewed in Gulbransen and Sharkey, 2012; Neunlist et al., 2014; Coelho-Aguiar et al., 2015; Sharkey, 2015). Ablation and functional inhibition of EGCs lead to severe GI inflammation (Bush et al., 1998) and decreased gut motility (Nasser et al., 2006), respectively. Indeed, EGCs respond to neuronal activity during bowel movement (Broadhead et al., 2012) and actively participate in the regulation of intestinal transit (McClain et al., 2014). EGCs also affect enterocytes and epithelial permeability by releasing factors such as *S*-nitrosoglutathione (GSNO) (Savidge et al., 2007), glial-derived neurotrophic factor (Zhang et al., 2010), 15-deoxy-Δ^12,14^-prostaglandin J2 (Bach-Ngohou et al., 2010), and pro-epidermal growth factor (Van Landeghem et al., 2011). This functional connection between the ENS and intestinal epithelium has been termed the neuronal-glial-epithelial unit to acknowledge its importance in GI health and disease (Neunlist et al., 2013). Finally, EGCs directly regulate inflammatory processes using immunological signaling pathways activated via, e.g., the major histocompatibility complex class II (Geboes et al., 1992), interleukin 1 receptor (Stoffels et al., 2014), chemokine CCL20 (Fagbemi et al., 2013), or toll-like receptors 2 and 4 (Brun et al., 2013; Esposito et al., 2014). Since ASD patients have increased epithelial leakiness and inflammation in the gut, as well as impaired bowel motility, EGCs could play an important role in ASD etiology and/or progression.

EGCs morphologically and functionally resemble astrocytes of the CNS (reviewed in Gulbransen, 2014) and altered expression of astrocytic markers was found in brains of autistic patients (Fatemi et al., 2008). One particularly interesting finding is the increased expression of connexin 43 (Cx43) in the superior frontal cortex, a part of the brain responsible for higher social, cognitive and emotional functions (Fatemi et al., 2008), suggesting that selective astrocytic network dysregulation plays a role in characteristic symptoms of autism. Additional work is needed to determine whether subcellular localization of Cx43 and functional cell-to-cell coupling via Cx43 are also affected. Nonetheless, the increased expression of Cx43 could be compensatory due to the protein removal from the cell surface. Here, internalization of Cx43 is quite possible because brain inflammation was recently recognized as a hallmark of autism (Gupta et al., 2014) and, concurrently, CNS infection and inflammation are known to cause Cx43 internalization in astrocytes (reviewed in Castellano and Eugenin, 2014).

Since the expression of Cx43 has been recently confirmed in EGCs (McClain et al., 2014), here, we speculate that changed expression of Cx43 in EGCs could affect the above mentioned ASD-related GI pathophysiology, i.e., motility, epithelial leakiness and inflammation. To corroborate our theory, we use findings from studies conducted on EGCs, as well as on astroglia. However, it is important to keep in mind that astrocytes and EGCs are two distinct cell types due to different origin and molecular composition (reviewed in Gulbransen, 2014).

Recent study showed the role of EGCs' Cx43 hemichannels in the regulation of the GI motility (McClain et al., 2014). Selective inhibition of Cx43 hemichannels reduced the Ca^2+^ wave propagation between the EGCs *in situ* and inducible knock-out of Cx43 encoding gene selectively from EGCs reduced the colonic transit *in vivo*. This genetic manipulation also reduced contractility of *ex vivo* colonic muscle strips. It is therefore tempting to speculate that constipation and reduced GI motility in ASD patients could partly come from the reduced Cx43 expression/function at the plasma membrane of EGCs. One possibility is a change in Cx43 transcription. For instance, some monogenetic ASDs that are caused by the mutations in genes encoding epigenetic or transcription factors, e.g., methyl CpG binding protein 2 (MeCP2) in Rett Syndrome (Yasui et al., 2013) or the above mentioned TCF4 in PTHS, respectively, could affect transcriptional machinery required for adequate expression of Cx43 in EGCs (reviewed in Oyamada et al., 2013) and, in turn, affect GI motility in patients with ASD. The other scenario could be the secondary effect of intestinal inflammation, a common GI feature in autistic patients, affecting Cx43 subcellular localization and function in EGCs.

Concurrently, Cx43 on EGCs is taking part in the inflammatory process. Neuronal loss is one of the characteristics of intestinal inflammation and is driven by the activation of neuronal purinergic receptor (Gulbransen et al., 2012). Recently, inhibition or genetic ablation of EGCs' Cx43 prevented the inflammation induced neuronal death (Brown et al., 2015). This is interesting because ATP released from EGCs via Cx43 hemichannels is involved in both inflammation and motility (McClain et al., 2014), as mentioned above. Additionally, direct cell-to-cell contact via Cx43 gap junctions can modulate immune responses (Westphalen et al., 2014), so EGCs might regulate immune cells activation in a similar fashion and further regulate the inflammatory process.

Animals with ablated Cx43 in EGCs also exhibited an increased fluid content in stools, indicating disabled water reabsorption/secretion (McClain et al., 2014). This could occur via altered EGC-enteroendocrine cell interactions (Bohorquez et al., 2014). Alternatively, the finding may imply a role of EGCs' Cx43 in regulation of the gut epithelium barrier, as EGCs are active players in the neuronal-glial-epithelial unit and have protective effects on enterocytes. A co-culture study showed that EGCs increase the transcription of enterocyte genes involved in cell-to-cell and cell-to-matrix adhesion, and also demonstrated an increase in cell adhesion (Van Landeghem et al., 2009). Some of the glia-derived factors, e.g., prostaglandins, could be released through the Cx43 hemichannels (Cherian et al., 2005). The other effects could come from the direct cell-to-cell contact. EGCs are in proximity of enterocytes (Liu et al., 2013) and both the cell types express Cx43 (Leaphart et al., 2007), so homotypic Cx43 gap junctions could couple these two cell types. Cx43 is known to regulate cell-to-cell contact gene expression (Lecanda et al., 1998), perhaps by affecting intercellular diffusion of secondary messengers and cytosolic Ca^2+^oscillations that can consequently regulate gene transcription (Dolmetsch et al., 1997; Sassone-Corsi, 1998). Additionally, gap junction coupling could also affect the membrane potential, due to the potential difference between the EGCs and enterocytes, i.e., about −55 mV and −25 mV (Cremaschi et al., 1982; Hanani et al., 2000), respectively, that could consequently also affect gene expression and cell physiology. Indeed, membrane potential of differentiating enterocytes becomes more positive exclusively due to their migration away from the crypt-villus junction (Cremaschi et al., 1984), where there is higher probability to interact with EGCs (Liu et al., 2013).

EGCs are also in proximity of capillaries within the gut wall (Fu et al., 2013), so they might have a role in the regulation of vascular permeability, resembling astrocytes that take part in the formation of the blood-brain-barrier (BBB). Indeed, EGCs have the intrinsic ability to participate in BBB formation after their transplantation into the spinal cord (Jiang et al., 2005). In the CNS, astrocytic specialized processes, termed endfeet, highly express Cx43 and wrap blood vessels (Simard et al., 2003). The loss of Cx43 causes astrocyte endfeet edema and weakens the BBB (Ezan et al., 2012). If similar mechanism takes place in the gut, the loss of Cx43 from EGCs would increase the permeability of the intestinal vasculature providing a link for the gut-brain axis in ASD, perhaps by contributing to brain inflammation (Gupta et al., 2014), and enhancing the CNS-related symptoms in autistic patients.

In summary, we presented a hypothetical link between the Cx43 of EGCs and GI related symptoms in patients with autism (Figure 1). EGCs interact with multiple cell types in the gut wall, perhaps by releasing factors via Cx43 hemichannels and/or cell-to-cell coupling via Cx43 gap junctions (Figure 1A). The changes in Cx43 expression, subcellular localization and function have the potential to directly affect motility, epithelial permeability, and inflammatory processes of the gut. These processes are connected in the feed-forward loop forming a vicious cycle (Figure 1B). Cx43 in EGCs could also affect the permeability of the intestinal vasculature and consequently contribute to the severity of ASD in the brain. At this juncture it is speculative whether GI manifestations are simply part of the ASD clinical picture or insult to the gut precedes autistic behavior. Since ASD is so diverse, both the scenarios may play out, but in different cases. EGCs and Cx43 could have a role in either of the scenarios and therefore deserve additional attention. This could eventually enable us to ameliorate, if not prevent, the GI disturbances and some of the CNS-related symptoms in patients with ASD.

**Figure 1:**
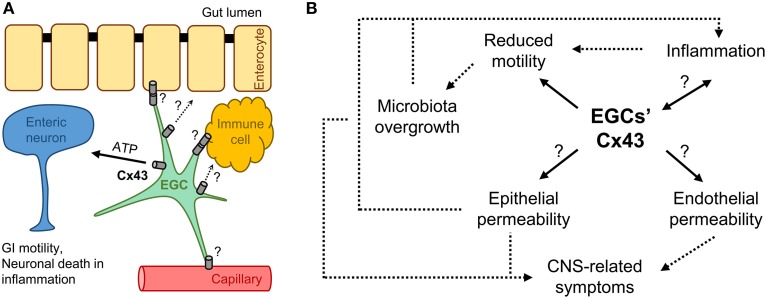
**Is Cx43 on enteric glia involved in autism spectrum disorder (ASD)? (A)** Enteric glial cells (EGCs) interact with enteric neurons within the enteric nervous system and other cells in the gut wall, such as enterocytes, vasculature endothelial and immune cells, regulating gastrointestinal (GI) motility, as well as epithelial/endothelial barriers and inflammation in the intestine; these interactions could happen through cell-to-cell coupling via Cx43 gap junctions or factors released through Cx43 hemichannels. **(B)** Possible roles for EGCs' Cx43 in the vicious cycle of GI disturbances in ASD. Regardless of the etiology, e.g., gene mutation or environmental factors, ASD often leads to similar GI clinical manifestations, i.e., reduced motility and inflammation, because pathophysiological processes are interconnected. Cx43 on enteric glia could be involved in all the processes and contribute to the overall clinical picture. Question marks (?) indicate untested interactions/processes. Arrows indicate relationships between the processes (dashed lines indicate processes not directly linked to EGCs' Cx43).

## Conflict of interest statement

The authors declare that the research was conducted in the absence of any commercial or financial relationships that could be construed as a potential conflict of interest.
